# Double laterally rotated bilayer flap operation for treatment of gingival recession: A report of two cases

**DOI:** 10.4103/0972-124X.44093

**Published:** 2008

**Authors:** Vijayaraghavan Anita, Rajaram Vijayalakshmi, J. Bhavna, Thyagarajan Ramakrishnan, Aravindkumar, Vikram Bali

**Affiliations:** Department of Periodontics, Meenakshi Ammal Dental College, Chennai - 600 095, India

**Keywords:** Attached gingival, pedicle flap, root coverage

## Abstract

Esthetic concerns of the patient have become an essential part of dentistry, especially Periodontics. Periodontal plastic surgery is a rapidly emerging field, which helps us to meet this criterion. Root coverage is being achieved by a variety of techniques namely pedicle grafts and free soft tissue grafts. This article highlights on 2 case reports in which a new pedicle graft technique has been used for root coverage.

## INTRODUCTION

Marginal tissue recession can cause major functional and esthetic problems. It has been clinically related to higher incidence of root caries, attachment loss, hypersensitivity and smile related concerns. The etiologic factor for gingival recession are high muscle attachments, frenal pull, iatrogenic factors, hard tooth brushes, traumatic tooth brushing and tooth malposition. The sequelae of gingival recession consists of a non keratinized tissue not firmly bound to the underlying periosteum and ill designed to withstand daily insults of toothbrushing and masticatory forces.[1]

The literature has documented that gingival recession can be successfully treated by means of several mucogingival surgical approaches irrespective of the technique utilized, provided that the biologic conditions for accomplishing root coverage are satisfied with no loss of soft and hard tissue height interdentally.

A graft is a piece of living tissue or synthetic material placed in contact with injured tissue to repair a defect or correct a deficiency.[1] There are currently different techniques for root coverage which includes pedicle grafts, free gingival grafts, connective tissue grafts and Guided tissue regeneration (GTR). Grupe and Warren *et al*, proposed the technique of laterally repositioned flap operation for coverage of isolated recessions.[2] The indications of laterally positioned flap are the presence of sufficient width, length and thickness of keratinized tissue adjacent to the area of gingival recession.[3] This method is most suitable for root coverage in gingival recession with narrow mesiodistal dimension. Guinard and Caffese reported an average of 1 mm of post operative recession on the adjacent donor site.[4] The disadvantages of this method are possible bone loss and gingival recession in donor site.

Many modified methods have been developed to avoid gingival recession in the donor site. Staffelino advocated the use of partial thickness flap to avoid recession on the donor site.[5] Grupe reported a modified technique to preserve the marginal gingival by making a sub marginal incision on donor site.[6] Pfeifer and Heller reported that reattachment to the exposed root surface is more likely to occur with full thickness laterally positioned flaps.[7] Ruben ***et al.*** demonstrated the partial and full thickness pedicle flap. The full thickness flaps are appropriate for root coverage and partial thickness flaps are suitable to protect the exposed roots.[8] Espinel and caffesse compared the full thickness and partial thickness pedicle flap.[9] Neken * et al*proposed that mean percentage of root coverage using laterally repositioned flap ranges from 34-82 %. The advantages of laterally repositioned flap over other flap procedure are the presence of its own blood supply after the transfer of the graft and high survival rate on the roots.

The goal of the present study was to evaluate the effectiveness of a modified surgical approach to laterally repositioned flap for coverage of isolated recession.

## METHODS

### Patient selection and pre-surgical preparation

Two patients came to the department of the Periodontology, Meenakshi Ammal Dental College for the treatment of gingival recession in relation to lower anteriors. Both patients were in good general health and had received no periodontal therapy during the previous 6 months. Prior to therapy data was obtained including dimensions of gingival recession, probing pocket depth and width of keratinized tissue. These measurements were made using a William′s periodontal probe. Clinical photograph was taken before and after the operation. The clinical parameters described were similarly recorded as indicated during follow up periods after surgery. Preoperative instructions included oral hygiene instructions, scaling and root planing and necessary plaque control schedules.

### Surgical technique

**Figure 1:**
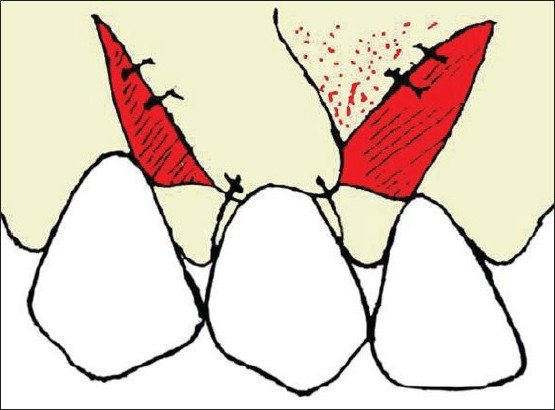
Horizontal incision at the interdental papilla and oblique releasing incisions at the proximal line angles of the teeth

Basically two partial thickness flaps were designed, one each on the mesial and distal sides of the gingival defect. Horizontal incision was made on the interdental papilla at the level of the CEJ. Two oblique releasing incisions were made at the proximal line angles of the adjacent teeth and extended beyond the mucogingival junction [Figure 1].

**Figure 2:**
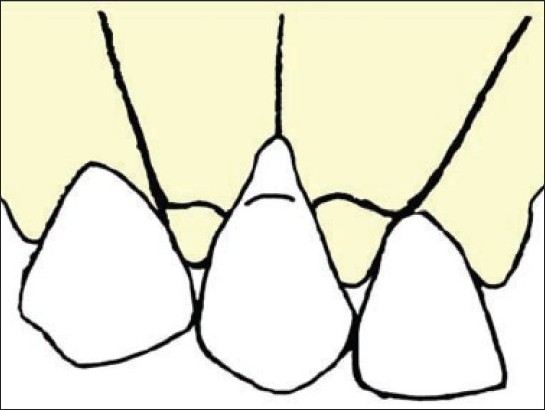
Partial - thickness flap elevated

**Figure 3:**
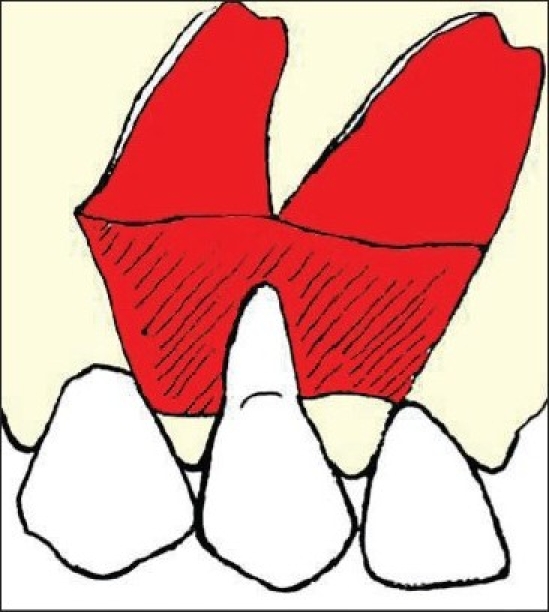
De-epithelialised mesial flap rotated to cover the recession

Both the mesial and distal portions of the partial thickness flap were elevated [Figure 2]. The exposed root surface was thoroughly planed with a curette. Then chemical root conditioning was performed using a cotton pellet soaked in citric acid solution pH-1 that was applied on the tooth surface for three minutes. The mesial or distal flap was de-epithelialized and the flap was rotated to cover citric acid conditioned root surface [Figure 3].

**Figure 4:**
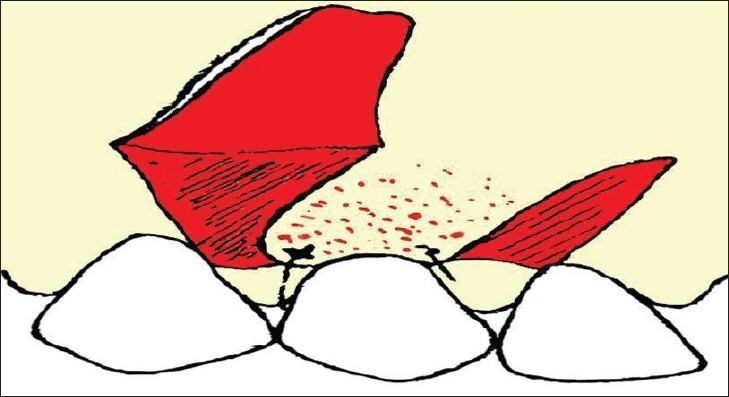
Unde-epithelialised distal flap rotated to cover the previous flap

The other un-deepithelialized pedicle flap was repositioned to cover the previous flap. The coronal margin of these two pedicle flaps was immobilized to the tooth using a sling suture [Figure 4].

The oblique releasing incisions on either side of the pedicle flaps were sutured onto the adjacent unreflected periosteum. A periodontal dressing was applied to the surgical site to protect the wound from irritation. After surgery, patients were instructed to discontinue tooth brushing at the surgical area for two weeks and to rinse with 0.12% chlorhexidine solution three times daily for 6-8 weeks. Amoxycillin 500 mg three times a day was prescribed for five days after surgery to prevent infection. Patients were recalled once a week for review for the first month.

The periodontal dressing was removed one week post-operatively. Post-operative follow-up visits were arranged at 1, 2, 3 and 4 weeks for three months. Postoperative measurements of clinical data were recorded at the end of six months after surgery. Regular maintenance care by scaling and plaque control was performed.

## CASE REPORT

### Case-1

A 24/M patient came to the department of Periodontics, Meenakshi Ammal dental college for treatment of gingival recession (Millers class-1) in relation to buccal side of 41.

**Figure 5:**
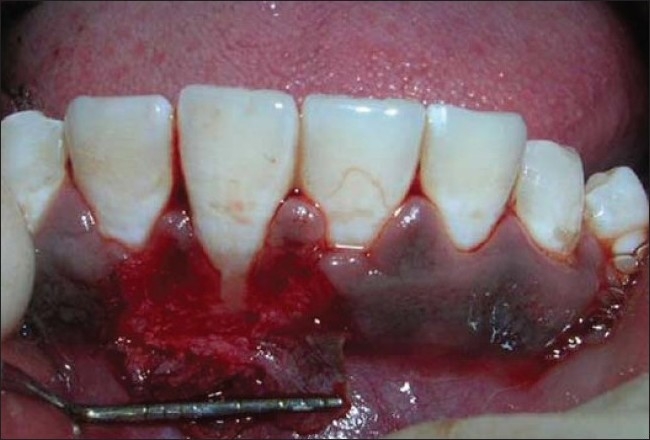
Pre-operative view of 41

On clinical examination the dimensions of gingival recession was 2mm in width and 2 mm in depth. The width of the keratinized gingiva adjacent to the area of recession was 3mm; probing depth of the affected tooth was 2mm [Figure 5].

**Figure 6:**
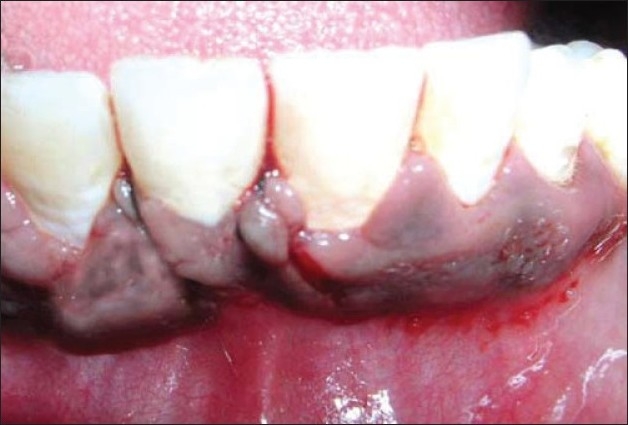
Horizontal incision at the interdental papilla and oblique releasing incisions at the proximal line angles of 41 placed

**Figure 7:**
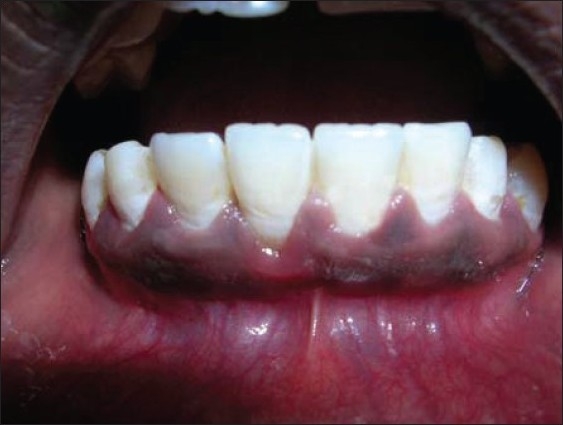
Partial - thickness flap elevated

**Figure 8:**
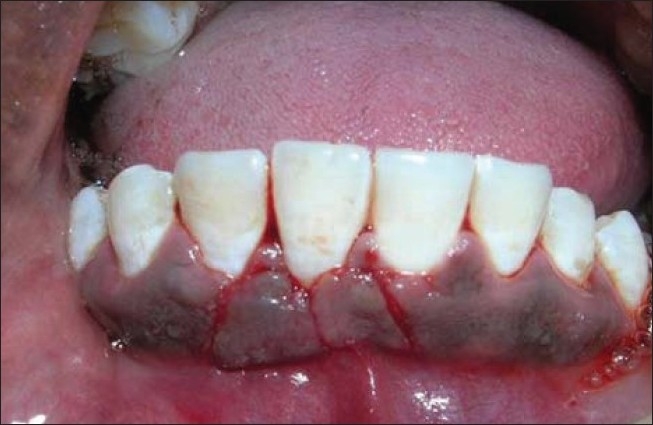
Flaps sutured to cover the recession in 41

After scaling and root planing procedures, the double laterally rotated bilayer flap operation was performed in relation to 41 [Figures 6–8]

**Figure 9:**
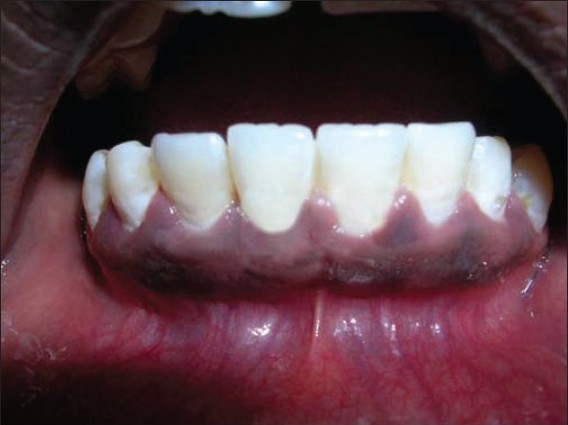
Post-operative view

The postoperative course was uneventful. The recession was completely covered and the width of the keratinized gingiva increased to 4mm at 6 months after surgery [Figure 9].

Regular checkups demonstrated that root coverage and width of the keratinized gingiva remained stable at 6 months. The patient maintained good oral hygiene and the probing depth in the midfacial surface of the treated tooth was normal (2mm) throughout the 6 months follow-up period.

### Case-2

The second patient was a 36/M who was treated for Miller's class-II gingival recession and tooth hypersensitivity in relation to 41.

**Figure 10:**
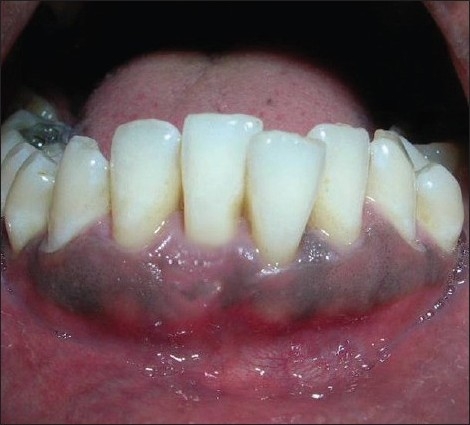
Pre-operative view of 41

The dimensions of gingival recession were width and depth of 3mm.The width of keratinized gingiva was 2mm. The probing depth of the affected tooth was 3mm [Figure 10].

**Figure 11:**
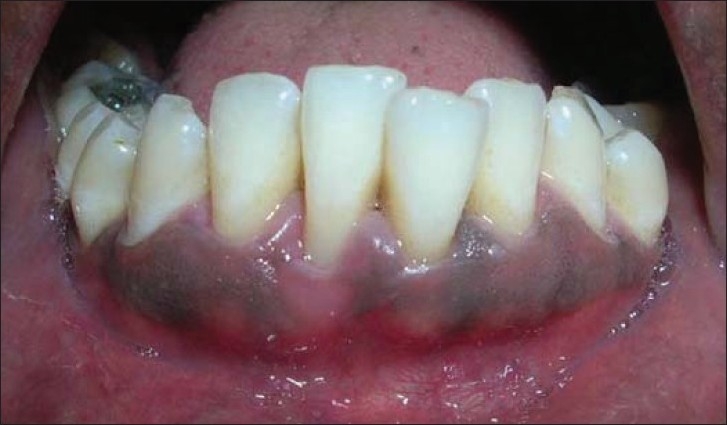
De-epithelialised distal flap rotated to cover the recession in 41

**Figure 12:**
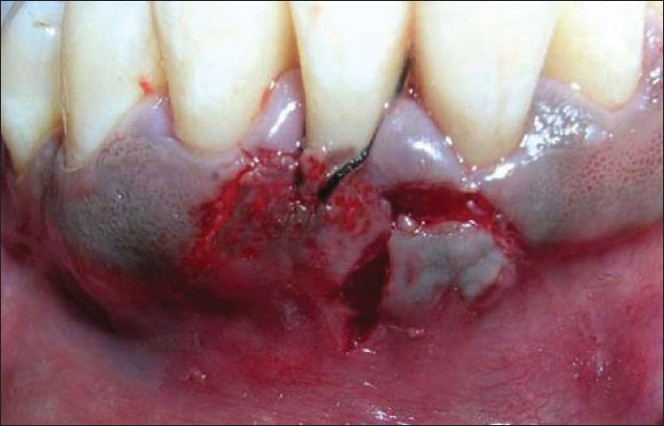
Unde-epithelialised mesial flap rotated to cover the previous flap

**Figure 13:**
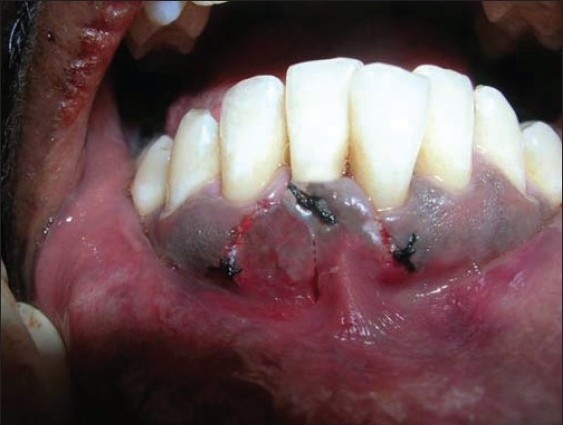
Post-operative view

Similar surgical technique was performed in relation to 41 [Figures 11 and 12]. Two weeks after surgery, satisfactory root coverage (3mm) with reduction in tooth hypersensitivity was found. The results remained unchanged during clinical follow-up of 6 months [Figure 13].

## DISCUSSION

Complete root coverage is the primary objective to be accomplished when treating gingival recession in patients with aesthetic demands. Many root coverage procedures have been widely accepted in clinical dental practice like GTR, free gingival grafts, connective tissue grafts, etc. Grupe and Warren in 1956 suggested that laterally repositioned flap has shown to be the most successful procedure for the treatment of gingival recession.[6]

This double laterally rotated bilayer flap technique has been demonstrated to be a reliable and predictable treatment modality for obtaining root coverage in an isolated gingival recession.

The advantages of this technique are reduced hypersensitivity, esthetic color matching, good blood supply to the reflected flap with high mean percentage of root coverage.[10] These root coverage outcomes were associated with significant clinical attachment gain with reduction in probing pocket depth. The root coverage was obtained with no change in the position of the gingival margin lateral to the defect.[11]

However there are still many limitations, which need to be considered when this technique is applied. These include the following:-

1. The interdental papillary tissue adjacent to the area of the recession should be thick, since a cause-effect relationship has been reported to exist between flap thickness and recession reduction.
2. There should be no deep periodontal pockets and bone loss beyond the mucogingival junction at the interdental areas of the affected tooth.
3. Separate surgical procedures are still needed in presence of multiple adjacent recessions.

In the present study, a new modification to laterally repositioned flap was introduced which has the advantage of both laterally positioned pedicle graft and a subepithelial connective tissue graft modified by Nelson *et al.*[12]

The analysis of the literature revealed that the limiting condition for performing this approach, as a root coverage surgical procedure was the need for adequate keratinized tissue lateral to the recession defect. Previous studies did not quantify the minimum width and height of keratinized tissue, which must be present lateral to gingival defects in order to render this approach a predictable root coverage surgical procedure. A certain amount of the lateral keratinized tissue, infact must be preserved in situ to prevent gingival recession at the donor site, while the remaining part is used to cover the exposed root surface and the adjacent connective tissue beds.[13]

This present study demonstrated that the proposed modification of the laterally displaced flap is an effective treatment modality for the management of recession defects affecting teeth in esthetic regions of the mouth. Infact this surgical technique resulted in complete root coverage in the treated cases.
